# Functional genetics – an approach to mechanisms in Alzheimer’s disease

**DOI:** 10.1186/1750-1326-8-S1-O3

**Published:** 2013-09-13

**Authors:** Mikko Hiltunen

**Affiliations:** 1 Institute of Clinical Medicine – Neurology, University of Eastern Finland and Department of Neurology, Kuopio University Hospital, 70211 Kuopio, Finland

## Background

Alzheimer’s disease (AD) is the most common neurodegenerative disorder in the world. As the aging population continues to increase globally, treatment of AD and other age-associated neurodegenerative diseases is becoming increasingly important, not only from a human point of view, but also from an economic perspective. In recent years, several attempts have been made to find novel susceptibility genes and pathways relevant for AD. Particularly genome-wide association (GWA) and meta-analysis-based studies have identified several risk variants in different genes, which significantly associate with AD. The subsequent mechanistic characterization of the identified risk genes is extremely important as it may pave the way for the development of new biomarkers and intervention approaches.

## Materials and methods

Microarray-based global expression and splicing analysis of AD brain samples (inferior temporal cortex) was conducted to identify novel AD-associated target genes and pathways. Brain samples were divided into mild, moderate and severe AD subgroups according to the neurofibrillary tangle (NFT) pathology (Braak staging) and expression and splicing changes were analyzed in relation to the disease severity. Furthermore, the most prominent risk gene variants identified in the recent GWA and meta-analysis-based studies were genotyped and used to elucidate the expression and splicing changes with respect to the risk gene profiles. α-, β-, and γ-secretase activity as well as soluble β-amyloid (Aβ) 42 measurements were performed from the same tissue samples and used for correlation analyses. A subset of the identified AD-associated target genes were further directed to high-throughput RNA interference (RNAi) *in vitro* screening to characterize specific target genes affecting amyloid precursor protein (APP) processing and Aβ generation.

## Results

Apart from the already established target genes known to be differentially expressed in the AD brain, several novel targets that showed altered expression and/or splicing with respect to disease severity were identified. Some of these novel targets revealed significant changes only in the late phase of the AD (Braak stages: V-VI), such as clusterin and complement factors. Several others were significantly associated with the NFT and Aβ42 changes already in the early phase of the disease (Braak stages: III-IV) and the differential expression and/or splicing was further strengthened at the later phases of the disease (Braak stages V-VI). Only a small portion of the identified AD-associated target genes revealed changes in the APP processing and Aβ generation after the RNAi-mediated down-regulation of target gene products in both non-neuronal and neuronal cell lines over-expressing APP. Detailed characterization of the target genes affecting APP processing and Aβ generation are currently underway both in *in vitro* and *in vivo* models of AD to unravel the underlying molecular mechanisms. Furthermore, the relationships between AD-associated risk gene profiles and the expression and splicing of specific targets as well as biological pathways and gene clusters are currently being assessed.

## Conclusions

It is expected that the functional genetic approach adopted here identifies specific molecular targets in AD pathogenesis that underlie its clinical manifestations. It is also likely that new biomarkers will be identified for risk assessment, early diagnosis, and disease progression.

